# Cardiovascular and Mortality Risks in Migrant South Asians with Type 2 Diabetes: Are We Winning the Battle?

**DOI:** 10.1007/s11892-017-0929-5

**Published:** 2017-09-18

**Authors:** Emma Johns, Naveed Sattar

**Affiliations:** British Heart Foundation Glasgow Cardiovascular Research Centre, University of Glasgow, 126 University Place, Glasgow, G12 8TA UK

**Keywords:** Diabetes mellitus, Type 2 diabetes mellitus, Cardiovascular risk, South Asian

## Abstract

**Purpose of Review:**

We seek to describe the relationship between diabetes mellitus and cardiovascular risk in migrant South Asians compared to native white Europeans, and to determine the temporal change in this relationship over recent years.

**Recent Findings:**

Recent evidence suggests that the excess mortality risk associated with diabetes is lower in the migrant South Asian population compared with white Europeans. By contrast, South Asians continue to demonstrate elevated cardiovascular morbidity compared to white Europeans, although to a lesser extent than was observed in previous decades.

**Summary:**

The excess mortality previously observed in South Asian migrants has attenuated with a lower mortality risk compared to white Europeans observed in several recent studies. We speculate that these findings may relate in part to earlier diabetes diagnosis and more prolonged exposure to cardiovascular risk factor management in the South Asian population. Further study is required to confirm these hypotheses.

## Introduction

Diabetes mellitus confers an approximately two-fold increased risk of cardiovascular morbidity and mortality relative to non-diabetes [1]. South Asians, particularly South Asian migrants to high-income countries, have a significantly increased risk of type 2 diabetes compared with white Europeans. Population level data from the end of the twentieth century suggested that South Asian migrants with diabetes mellitus were at increased risk of complications, both cardiovascular disease (CVD) and cardiovascular death, relative to their white European counterparts [2–4]. However, some recent evidence suggests there is now less marked disparity in some cardiovascular complications between these populations, with additional surprising evidence of a lower mortality risk compared to white Europeans [5]. Our aim is to review the literature in this field and examine evidence for a temporal change in these relationships. We will also speculate on the mechanisms behind such changes.

## Diabetes Mellitus in Migrant South Asians

There are several million migrant South Asians, including Indian, Pakistani, Bangladeshi and Sri Lankan individuals, living outside the Indian subcontinent. The estimated population size includes 3 million people in the UK, 3 million people in the USA and 1.6 million people in Canada [6••]. The prevalence of type 2 diabetes mellitus is higher in these populations compared to the native white populations in the countries to which they move [2, 7–9]. In the UK, this pattern was first formally described in the 1985 Southall Diabetes Survey [2]. This community survey of over 65,000 participants in west London showed a 5-fold higher prevalence of diabetes mellitus in South Asian residents aged 30 to 64 compared to their white European neighbours. Modern estimates are more modest but show the risk remains elevated by approximately 2 to 4-fold in migrant South Asians compared to white Europeans [7, 8]. The magnitude of this risk is variable within the South Asian population with the highest risk seen in the Bangladeshi community (approximately 4-fold) compared to a roughly 2-fold risk in the Indian community [8, 9]. The reasons behind this intra-ethnic heterogeneity in risk are unknown but require further study.

## Aetiology of Diabetes and Cardiovascular Risk in South Asians

The onset of type 2 diabetes typically occurs 5 to 10 years earlier in South Asians and the risk begins to increase at far lower levels of BMI compared with white Europeans [7, 10]. The increased risk of diabetes is likely to relate to multiple intrinsic and environmental factors. Several potential mechanisms were recently reviewed in detail by Sattar and Gill and these are summarised in Table 1 [6••].

**Table 1:** Mechanisms potentially underlying the higher risk of type 2 diabetes mellitus in South Asians

Mechanism	Evidence
Adiposity	At a given BMI, South Asians have a higher fat mass and a larger proportion of fat in deep abdominal subcutaneous and visceral deposits compared to white Europeans. There is also some evidence for greater liver fat. Such findings are in keeping with greater insulin resistance in South Asians
Lean body mass	South Asians have less lean tissue than white Europeans for any given BMI. This relative lack of skeletal muscle and capacity for glucose disposal contributes to greater insulin resistance and diabetes risk
ß-cell function	Compared to white Europeans, South Asians have greater ß-cell function until adulthood but earlier and more rapid decline in insulin production beyond middle age. Such evidence requires verification using robust measures of beta-cell function
Fitness and skeletal muscle function	South Asians obtain a lower level of cardiorespiratory fitness than white Europeans for the same level of physical activity and have evidence of reduced fat oxidation (indicative of impaired muscle metabolism) during submaximal exercise compared with BMI-matched white Europeans. Once again, studies using more robust measures of muscle function are required to verify these findings
Lifestyle	South Asians are less physically active than white Europeans. There is no consistent evidence linking South Asian dietary patterns to increased diabetes risk; however, short term overfeeding may have greater adverse effects in South Asians
Genetic factors	There is some evidence that epigenetic signals may contribute to the increased risk of diabetes in South Asians. These signals appear to relate mostly to impaired beta-cell function. Genetic risk factors for diabetes do not appear to significantly differ between South Asians and white Europeans
Foetal programming	There is little robust evidence for foetal programming underlying the excess risk of diabetes in South Asians; observational data suggests that increased maternal fasting blood glucose levels mediate the relationship between South Asian ethnicity and greater fat mass in offspring but the long-term implications of this finding is not clear

(Adapted from: Sattar N, Gill JMR. Lancet Diabetes Endocrinol. 2015;3(12):1004–16, with permission from Elsevier) [6••]

## Cardiovascular Morbidity

The impact of South Asian ethnicity on risk of diabetes-related complications has been examined in several population-based studies. However, the nature of the relationship remains uncertain. The 11-year follow-up of the Southall Diabetes Study, performed in 1995, showed that amongst the 590 surviving diabetic patients, the rate of previous myocardial infarction was almost four times higher in South Asians compared with white Europeans [11]. However, there was no significant difference observed in the risk of hypertension, stroke or amputation. The SABRE cohort presented 20-year follow-up data from patients initially recruited to the Southall study between 1988 and 1991 [3]. The risk of stroke was almost twice as high in South Asians with diabetes compared with their white European counterparts and the risk of coronary heart disease was also elevated to a lesser extent (subhazard ratio 1.55; 95% CI 1.38–1.75). The UK Asian Diabetes Study collected data between 2004 and 2007 from 1486 patients diagnosed with diabetes in the mid-1990s. This study showed a trend towards increased risk for cardiovascular events or death from CVD in South Asians compared with white Europeans; however, this was not statistically significant (adjusted odds ratio [OR] 1.4; 95% CI 0.9 to 2.2) [12]. A large Canadian population-based study of newly diagnosed diabetic individuals from 2002 to 2009 included almost half a million participants, 22,432 of whom were South Asian [5]. Over a 5-year follow-up period, the risk of hospitalisation for coronary artery disease, stroke or lower-extremity amputation was determined to be broadly similar for South Asian and white European participants (hazard ratio [HR] 0.95; 95% CI 0.9–1.00). The UK National Diabetes Audit 2010–11 included data from over 2 million patients representing 87.6% of persons with diabetes in England and Wales [13]. The risk of several cardiovascular outcomes was elevated in South Asians compared to white Europeans: myocardial infarction (OR 1.6; 95% CI 1.5–1.7), angina (OR 1.2; 95% CI 1.18–1.3), heart failure (OR 1.3; 95% CI 1.2–1.34) and stroke (OR 1.08; 95% CI 1.01–1.2). A Canadian population-based study examined incidence of macrovascular complications in over 250,000 newly diagnosed persons with diabetes from 1997 to 2007 [14]. In this cohort, South Asian and white European women had a similar risk of stroke, myocardial infarction and heart failure. For South Asian men, the risks of heart failure (HR 0.71; 95% CI 0.56–0.92) and stroke (HR 0.82; 95% CI 0.68–0.99) were lower compared to white European men, whilst the risk of myocardial infarction was similar. However, this study did not adjust for important confounders such as smoking and BMI; therefore, some caution is needed in the interpretation of the apparent reduced risk of these outcomes.

Nevertheless, taken together these studies broadly support somewhat higher risks of cardiovascular complications in South Asians with diabetes relative to their European counterparts but risk differences being appreciably lower than seen a few decades ago. There is however some heterogeneity of risk levels both between studies and by the nature of the outcomes examined. Some of these differences may arise from differential adjustment for confounders and thus further contemporary and well-designed studies are required in this area. Such studies conducted in high-income countries would be usefully complemented by studies in low and middle-income countries, although one accepts the technical challenges in the robust capture of data and outcomes in less affluent countries.

## Cardiovascular Mortality

As with CVD risk in general, South Asian ethnicity has been shown to impact the relationship between type 2 diabetes and mortality, although the nature of this impact is somewhat uncertain and appears to have meaningfully changed. An increased risk of mortality from CVD in South Asians with type 2 diabetes was demonstrated in national mortality data from England and Wales collected from 1985 to 1986 [4]. Compared to the native white European population, South Asians demonstrated increased mortality across the spectrum of cardiovascular death. Rates of all-cause mortality, cardiovascular mortality and stroke deaths were approximately twice that of Europeans and deaths from ischaemic heart disease almost three times greater. Relative age-specific mortality rates showed the ethnic difference to be greatest in the youngest age group, for example ischaemic heart disease death rates were four times higher in younger South Asians (45 to 64 years), compared with three times higher in those aged 65 and above. In the Southall 11-year follow-up cohort, collected in 1995, mortality relating to heart disease and circulatory deaths in patients aged 30 to 64 at baseline was approximately twice as high in South Asians compared to white Europeans [11]. For patients aged 65 and older from the outset of the study, mortality rates were similar.

By contrast, more recent studies suggest lower mortality risks in the South Asian diabetic population compared to their white European counterparts. In the previously mentioned study by Shah et al., involving incident cases of diabetes in Canada between 2002 and 2009, the all-cause mortality was more than 40% lower in South Asians compared to Europeans (HR 0.56, *p* < 0.001) over the 5-year follow-up period [5]. Additionally, the Canadian study following newly diagnosed diabetics over a 10-year follow-up period from 1997 to 2007 showed all-cause mortality was lower in South Asians than white Europeans (HR 0.69; *p* < 0.01) [14]. Likewise, the UK National Diabetes Audit 2010–11 showed all-cause mortality was almost 50% lower in South Asians compared with white Europeans (OR 0.53, 95% CI 0.50–0.56) [13]. The 2011–12 National Diabetes Audit provided estimates for type 1 and 2 diabetes and suggested a similarly lower risk of mortality in South Asians with type 2 (point estimate 0.53; 95% CI 0.5–0.55) and a more modest difference in risk in South Asians with type 1 (point estimate 0.75; 0.6–0.9) [15]. Furthermore, the 2012–2013 National Diabetes Audit showed that the risk of death from heart failure was in fact lower in South Asians with both type 2 (−35.2%; 95% CI −37.2 to 33.1%) and type 1 diabetes (−22%, 95% CI −35.2 to −6.1%) compared to white Europeans [16].

A recent UK population-based study—using the Clinical Practice Research Datalink (CPRD) cohort—went further and examined the relationship between ethnicity, life expectancy and cause-specific mortality for the first time [17••]. A total of 187,968 patients with incident type 2 diabetes between 1998 and 2015 were identified alongside 908,016 non-diabetic controls. The difference in life expectancy between diabetic and non-diabetic patients was found to be most pronounced within the white European population and least pronounced in the South Asian population. For example, in white Europeans at an attained age of 40 years old, diabetes was associated with a potential loss of life of 5 years in men and 6 years in women, findings in keeping with the *Emerging Risk Factors* publication a few years before [18]. This is compared to a potential loss of life of 3 years in South Asian men and less than 2 years in women. The difference in life expectancy comparing diabetic and non-diabetic individuals appeared greatest for both ethnicities aged 40 and declined with advancing age, in line with previous evidence that younger onset diabetes is potentially more harmful. Remarkably, however, diabetes was associated with a better life expectancy versus non-diabetic counterparts in older South Asians (greater than 60 years in women and 65 years in men). Mortality in diabetic South Asians was also generally lower relative to Europeans for a range of outcomes including all-causes (HR 0.70, 95% CI 0.65–0.76), CVD (HR 0.82, 95% CI 0.75–216 0.89) cancer (HR 0.43; 95% CI 0.36–0.51) and respiratory diseases (HR 0.6; 95% CI 0.48–0.76).

**Fig. 1:**
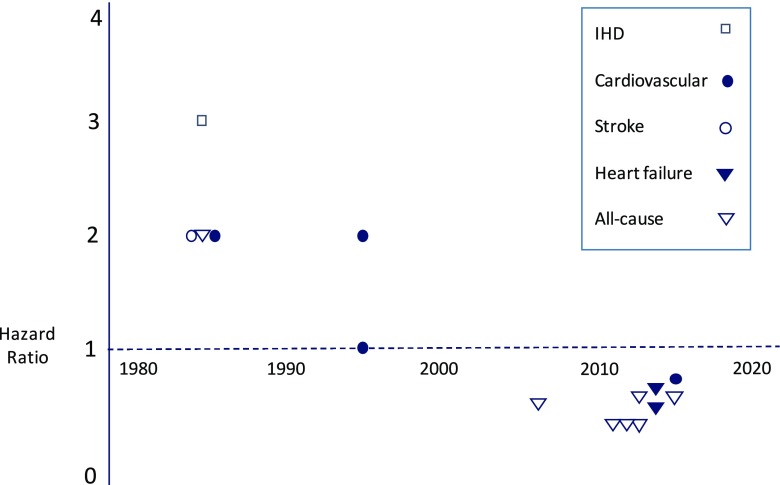
Relative risk of mortality in migrant South Asians with diabetes versus white Europeans reported in papers spanning the last 3–4 decades. All relevant studies discussed in the text [4, 5, 11, 13–16, 17••]. IHD ischaemic heart disease

The totality of data therefore suggests a beneficial shift in the pattern of cardiovascular mortality in South Asians with type 2 diabetes. Indeed, the previously observed excess cardiovascular and all-cause mortality observed in South Asians appears to have been reversed (Fig. 1).

## Speculating on the Mechanisms Underlying Changes in Mortality Risks

**Fig. 2:**
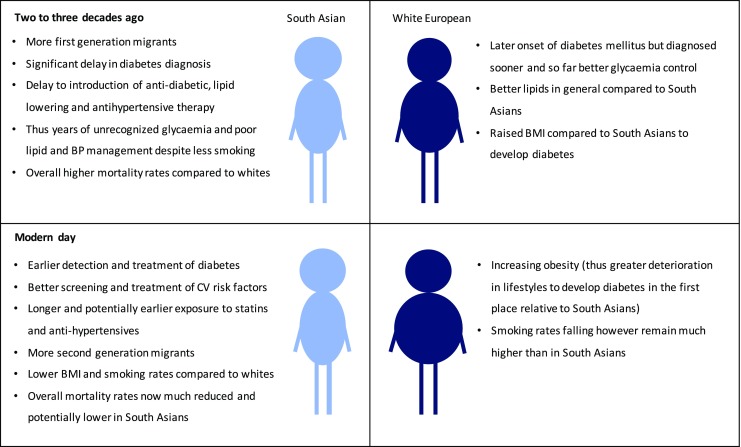
Speculated mechanisms for changing cardiovascular mortality risks in migrant South Asians with diabetes relative to white Europeans. Several proposed mechanisms require confirmation from future studies

So, what may be the mechanisms underlying such dramatic changes in mortality risk in migrant South Asians with diabetes? We suggest several factors may operate to explain these changes, as summarised in Fig. 2. We accept many of these are speculative and require direct confirmation but we believe our suggestions are potentially credible.

Over the past 20 years, patients with diabetes have benefited from the effective cardiovascular risk factor management with widespread provision of lipid-lowering and antihypertensive therapies. South Asians typically develop diabetes 5 to 10 years earlier than white Europeans and are therefore likely to benefit from earlier and more prolonged exposure to these agents, for example statins and ACE inhibitors. Although there is no firm evidence that statins offer a greater lipid-lowering effect to South Asians than white Europeans, this population does possess a more atherogenic lipid profile and therefore may accrue greater absolute benefit from long-term lipid-lowering therapy [19]. Interestingly, Khan et al. found that the lower mortality observed in South Asians compared to white Europeans persisted after adjustment for statin and ACE inhibitor prescribing, although such medications were only captured at one time point [14]. Other potential mechanisms could therefore operate, including earlier diagnosis of diabetes so that the time between true onset and doctor diagnosis may have been substantially reduced in recent years, perhaps particularly in South Asians. In this respect, earlier studies which reported high mortality in south Asians with diabetes will have included many more first generation South Asian migrants who would have had less stringent health care and longer periods of undiagnosed diabetes, with some having developed diabetes before they migrated. Consequently, South Asians with diabetes would have had much worse glycaemia control around two to three decades ago likely allied to poorer management of hypercholesterolemia and hypertension. Whilst glycaemia levels tend to still be somewhat worse in South Asians with diabetes relative to white Europeans, such differences are likely to have markedly reduced over the last few decades [20]. Of course, earlier pick up of diabetes allied to potentially earlier commencement of statins and blood pressure medications in South Asians (due to younger average age of onset), plus improved glycaemia management in general, predict that CVD risks (fatal and non-fatal) should have reduced markedly over the last few decades, as appears the case from the aforementioned review of the literature (Fig. 1).

Of course, the recent apparent lower, not higher, mortality in South Asians with diabetes cannot be due only to better glycaemia and lipid management in this ethnic group. We speculate lower exposure to smoking is relevant and that lower levels of obesity in South Asians with diabetes may also be highly relevant. That South Asians need to put on less weight to develop diabetes relative to their white counterparts means their lifestyles (calorie excess, activity levels) leading to obesity, as well as some of the downstream consequences linked to obesity (but independent of insulin resistance) such as hypertension must be less aberrant compared to their non-diabetic counterparts in comparison to the pattern seen in whites. In whites, individuals must put on more weight to develop diabetes and therefore their lifestyle must have undergone bigger deteriorations by the time diabetes develops. If true, such findings would potentially explain lower mortality from several diseases where obesity (and related lifestyle factors) are relevant and thus, not only deaths from CVD but also from cancer and respiratory causes might be lower, as appears to be the case [17••]. Of course, further verification of the recent CPRD findings would be welcome but it appears that at least in some high-income countries, CVD risks and related mortality in south Asians appear vastly reduced. Of course, we accept that some biases may exist such as the greater arrival of healthier migrants into high-income countries in recent years although the continued higher diabetes rates mean that this cannot be the only explanation.

## Conclusion

Our review of the literature suggests CVD risks in general and mortality risks more so have been markedly lowered in South Asians with diabetes in high-income countries and that more work should be done to confirm or refute our proposed mechanisms for this remarkable pattern. Finally, it is notable that such findings appear in stark contrast to the current patterns of CVD risk in low and middle-income South Asian countries, as recently reported [21]. Thus, whilst the battle to lower CVD risks is being won in migrant south Asian communities, it is only just beginning in many developing regions and there is much progress still to be made in this area worldwide.
